# A systematic review on whether regenerative agriculture improves animal welfare: A qualitative analysis with a One Welfare perspective

**DOI:** 10.1017/awf.2023.28

**Published:** 2023-04-25

**Authors:** Matías Javier Hargreaves-Méndez, María José Hötzel

**Affiliations:** Laboratório de Etologia Aplicada e Bem-Estar Animal (LETA), Departamento de Zootecnia e Desenvolvimento Rural, Universidade Federal de Santa Catarina. Rod. Admar Gonzaga, 1346, Itacorubi, 88034-001, Florianópolis, SC, Brazil

**Keywords:** Animal welfare, Causal Loop Diagram, livestock welfare, subjective well-being, sustainable agriculture, systems thinking

## Abstract

The welfare of animals in food-production systems is a cause of concern to the public. Regenerative agriculture was first used by the Rodale Institute and proposes to regenerate degraded components of ecosystems, aiming to be more than just sustainable. However, despite animal welfare being pushed to be part of the SDG agenda for 2030, there is no clarity on how regenerative agriculture impacts animal welfare. It is challenging to determine regenerative agriculture impacts on animal welfare, since it is not entirely defined. One Welfare could help define entry points for future research by studying animal welfare in connection with human welfare and environmental conservation. We aimed to analyse the extent to which positive animal welfare outcomes characterise regenerative agriculture systems in peer-reviewed articles and whether the narratives of such articles support that regenerative agriculture promotes animal welfare directly or indirectly by improving human welfare and environmental conservation. We searched papers including ‘regenerative agriculture’ using PRISMA-P, selecting animal welfare, human welfare, environment conservation terms, developed themes, and carried out analysis using Atlas.Ti8 and Causal Loop Diagram. We found that papers mainly linked animal welfare to animal health, human welfare to financial farm status and farmer’s self-awareness, and environmental conservation to soil improvement. Causal Loop Diagram indicated that regenerative agriculture had the potential to improve the health and nutrition components of animal welfare by enhancing financial farmers’ status/self-awareness (human welfare), and the soil (environmental conservation), reflecting that the processes that affect human welfare and environmental conservation could also affect animal welfare. However, information in papers remains insufficient to determine how regenerative agriculture impacts on animal welfare and research into regenerative agriculture needs to extend its focus on animal welfare and elucidate the regenerative agriculture principles leading to animal welfare.

## Introduction

By 2030 the global human population will have reached 8.6 billion people (United Nations 2019) and an estimated 815 million people are already prone to undernutrition in 2020 (Lal 2020a), a scenario that has been aggravated by the COVID-19 pandemic and climate change. Predicted numbers of refugees due to economic and climate change reasons are uncertain, with values between 50 and 250 million by 2050 (Burrows & Kinney 2016; Food and Agriculture Organisation of the United Nations 2021). There is a general need to revise our food production systems, seeking greater sustainability (Broom 2019), given that agriculture has been proven to be a significant contributor to exceeding planetary boundaries (Foley *et al.* 2005; Steffen *et al*. 2015; Campbell *et al*. 2017). Rhodes (2017) has explained that new alternatives for producing food should aim to actively regenerate ecosystems, instead of merely sustaining an ecosystem that may already be in a state of degradation.

Regenerative agriculture was coined by the Rodale Institute in the 1980s and regained popularity in 2016 amongst practitioners and scientists (Giller *et al*. 2021). Regenerative agriculture uses a systems thinking approach and proposes a set of principles that aim to restore the resource base of ecosystems and can help farmers to deal with complexity (Jones 2003; Mann *et al*. 2019). However, regenerative agriculture is mainly measured by the outcomes it generates, such as fertile soil, improved biodiversity, carbon sequestration, and other ecosystem indicators (Xu *et al*. 2019; Newton *et al*. 2020). This focus on outcomes has been challenging the research on regenerative agriculture, generating a lack of scientific consensus about the set of principles or processes that would lead to such outcomes. Some of these outcomes are more well-documented scientifically, particularly the ones related to soil improvement (Xu *et al*. 2019; Schreefel *et al*. 2020), and other outcomes, such as the ones associated with the social sciences, are gaining momentum in the regenerative agriculture literature (Gosnell *et al*. 2019; Brown *et al*. 2021; Gosnell 2021). While the Rodale Institute includes in their Regenerative Organic certification the improvement of soil health, human welfare and animal welfare (Alliance 2021), the information in scientific sources seems scattered and unclear. In their literature reviews, Giller *et al*. (2021) define regenerative agriculture as an approach aiming to combine agroecology and sustainable intensification to face land degradation, whereas Schreefel *et al*. (2020) define it as an approach that uses soil conservation as the starting point to regenerate and contribute to ecosystem services. Additionally, other authors described principles for regenerative agriculture, such as: (i) abandoning tillage; (ii) reducing spatial-temporal events of bare soil; (iii) enhancing soil fertility; (iv) diversifying cropping systems with livestock integration; (v) increasing biodiversity; (vi) increasing carbon sequestration; and (vii) reducing or eliminating synthetic agrichemicals (Rhodes 2017; Elevitch *et al*. 2018; LaCanne & Lundgren 2018; Newton *et al*. 2020; Luján Soto *et al*. 2021; Lundgren *et al*. 2021).

In the scientific literature, regenerative agriculture seems to focus on environmental conservation outcomes related to soil enhancement, biodiversity improvement, and increasing carbon sequestration (Xu *et al*. 2019; Newton *et al*. 2020; Giller *et al*. 2021), while there is a lack of mentions of animal welfare. A similar situation happens with sustainable agriculture, which has focused on its main components (environmental, economic, and social) leaving animal welfare components unattended (von Keyserlingk *et al*. 2013; Von Keyserlingk & Hötzel 2015). Moreover, a system could be considered sustainable when its present and future effects are acceptable to the general public (Broom 2016, 2017). Therefore, by neglecting animal welfare, both sustainable and ‘more than sustainable’ initiatives could compromise its social licence to operate since there is a growing public concern about farm animal welfare in food production systems (Clark *et al*. 2016; Cornish *et al*. 2016; Hötzel & Vandresen 2022). Regenerative agriculture, as a more-than-just-sustainable and incipient initiative, should address this gap and make explicit its impacts to the welfare of animals, and show evidence of the potential positive animal welfare, human welfare, and environment outcomes. Without this evidence, a system should not be considered regenerative (Alliance 2021). Broom (2021) proposed a method for assessing sustainability, finding that semi-intensive silvopastoral beef production systems are the more sustainable. These systems could not achieve this sustainability status without society’s acceptance, particularly concerning animal welfare. There are two main reasons for considering the impacts on animal welfare. Firstly, animal welfare has multiple relevant relationships with the Sustainable Development Goals from the United Nations (Keeling *et al*. 2019), and the scientific community is pushing governments to consider animal welfare as an integral part of these goals’ agenda for 2030 (e.g. Sebo *et al*. 2022). Secondly, regenerative agriculture can ensure public support. A recent study mentions animal welfare as a well-established on-farm benefit of regenerative agriculture (Spratt *et al*. 2021). While Spratt *et al*. (2021) do not provide methodological details about how to improve animal welfare comprehensively in regenerative agriculture, the mere mention of animal welfare reflects that the authors are giving a potential relevance to include animal welfare in regenerative agriculture studies.

Human welfare elements also seem understudied in the scientific literature about regenerative agriculture. Newton *et al*. (2020) found in journal articles that improved human health and profitability are possible outcomes of regenerative agriculture, but such articles provided no methodological details. According to the Human Development Index’s (HDI) dimensions from the United Nations (2020), human welfare is the capacity of a human to have a long and healthy life, knowledge, and a decent economic standard of living. Moreover, Diener *et al*. (2018) and Brown *et al*. (2021) describe that the assessment of human welfare should also consider measurements of subjectiveness, such as biological/temperament theories, satisfaction of goals theories, and mental-state theories for a more comprehensive understanding of human welfare.

One Welfare is a framework that can help determine how regenerative agriculture studies include the improvement of animal welfare in their narratives. Some studies might include elements of animal welfare explicitly (e.g. measurements or actions whose priority is to improve animal welfare), while others can include elements that could result in benefits for the animals, such as those related to human welfare and environment conservation. The One Welfare framework proposes that animal welfare should be studied from a systems-thinking approach in connection with human welfare and environmental conservation to achieve global-sustainable welfare (García Pinillos 2018). A systems-thinking approach could, firstly, uncover unseen relations between animal welfare, human welfare, and environmental conservation in regenerative agriculture narratives that could serve as entry points from where to start working on animal welfare goals. Secondly, it could identify how relevant papers documenting so-called regenerative agriculture are excluding fundamental elements for assessing animal welfare and human welfare.

The objective of this study was to analyse the extent to which positive animal welfare outcomes characterise so-called regenerative agriculture systems in peer-reviewed articles and whether the narratives of these articles support that regenerative agriculture promotes animal welfare directly or indirectly by improving human welfare and environment conservation.

## Materials and methods

### Inclusion of the One Welfare categories in peer-reviewed articles about regenerative agriculture

We searched peer-reviewed papers that included the words ‘regenerative agriculture’ from 1969 to 2021 to find inclusions of animal welfare, human welfare, and environmental conservation terms. We defined terms as any mention in the full text of the reviewed papers that combined empirical data, deductions from the authors, and potential outcomes (or benefits derived from processes) that the authors considered to be part of the impacts of regenerative agriculture. We did this to acknowledge that the literature is inconsistent in the usage of the word regenerative, and therefore whatever application of the concept needs to address a range of interpretations. In this study, we selected papers that explicitly mentioned regenerative agriculture in their full texts. To search and select the papers, we used the methodological framework PRISMA-P (Preferred Reporting Items for Systematic Reviews and Meta-analysis Protocols) (Pahlevan Sharif *et al*. 2019; Page *et al*. 2021). We used Scopus and Web of Science databases for regenerative agriculture in September 2021. The decision to use Scopus and Web of Science was based upon the higher number of results for ‘regenerative agriculture’ compared to other databases. To orient our search in the databases, we expanded the search words proposed by Schreefel *et al.* (2020): ‘([‘regenera* agri*’ OR ‘regenera* farm*’] OR [‘regenera* agro*’] OR [‘regenera* food system’] OR [‘regenera* and feed system’] OR [‘regenera* system’ AND agri*])’ (Schreefel *et al*. 2020). We added Holistic Management search words (‘Holis* manage*’ AND graz*). We set the databases’ configuration to look for the inclusion of these search words in the title, abstract and/or keywords of peer-reviewed articles. We decided to exclude the animal welfare, human welfare and environmental conservation terms from the search words to avoid limiting the number of papers. This decision was based on a previous search in the Scopus database using regenerative agriculture, animal welfare, human welfare, and environmental conservation search words together (in the title, abstract, or keywords), and finding only one eligible paper after screening. Thus, we searched for animal welfare, human welfare, and environment conservation terms within the regenerative agriculture papers’ full text for the qualitative analysis.

### Animal welfare

We divided the animal welfare information into categories to orient the search in the full-text screening, based on the Five Domains model adapted by Mellor *et al*. (2020). The animal welfare terms were divided depending on their relations to the domains Nutrition, Environment, Health, Behaviour, or Mental States. We decided to do this categorisation to determine if the animal welfare terms in the papers were focused on a specific domain. We used this model because it includes, in addition to the physical/functional domains (i.e. Nutrition, Environment, Health, Behaviour), elements to assess positive and negative affective experiences, which would expand the range of potential terms when searching in the full text. We associated the terms to the Five Domains, relating each term to the physical/functional and affective experience domains.

### Human welfare

The One Welfare framework defines and assesses the welfare of human and non-human animals in the same way, so measurements could be beneficial for both. In this study we used the terminology welfare, instead of well-being, when referring to human welfare, mainly because welfare and well-being concepts mean essentially the same and refer to individuals (Tarazona *et al*. 2020). We revised the human welfare information and classified it into terms. We used the Human Development Index’s (HDI) dimensions from the United Nations (2020), which describes three core dimensions for human welfare: long and healthy life, knowledge, and a decent standard of living. We complemented the HDI with the subjective well-being model by Diener *et al*. (2018), which uses theoretical processes to understand subjective welfare: biological/temperament theories, the satisfaction of goals theories, and mental-state theories. The biological/temperament theories explain how genetics influence that some people are happier than others, and the satisfaction of goals theories explain that people will be satisfied with their lives if their goals are completed. The mental-state theories describe cognitive and attentional processes that determine happiness depending on the individual’s perception and comparison with reference points. For example, Diener *et al*. (2018) showed an example of two people with the same income that could have different happiness levels depending on their previous economic standards and reference points. We associated the human welfare terms with the HDI dimensions and the subjective welfare model. Finally, we conducted our own interpretation of whether each term was ‘likely to improve human welfare.’ We did this by connecting the values from the HDI dimensions and the subjective welfare to a hedonic and eudaimonic subjective welfare. Hedonic subjective welfare relates to feeling pleasure, and it can be explained through emotional responses of people towards their own life, while eudaimonic subjective welfare focuses on how worthwhile people perceive their occupations to be (Brown *et al*. 2021). Then we created three classifications: (i) likely to improve human welfare; (ii) likely to reduce human welfare; or (iii) unclear.

### Environmental conservation

We oriented the full-text screening for environment conservation terms based on the One Welfare element ‘Livestock role in sustainable production’, and one of the principles of regenerative agriculture’s definitions: the integration of livestock (Rhodes 2017; Elevitch *et al*. 2018; LaCanne & Lundgren 2018; Lundgren *et al*. 2021; Luján Soto *et al*. 2021). As a result, we selected studies that: (i) included livestock; and (ii) mentioned a specific role of livestock in environmental conservation. We expected to find terms related to the livestock production impacts that are common in the literature, such as water pollution/utilisation and land utilisation (Broom 2019), carbon footprint and greenhouse gases emissions (Herrero *et al*. 2011; Cheng *et al*. 2022), carbon sequestration (Mosier *et al*. 2021), improved biodiversity (Gravuer *et al*. 2019), increased animal welfare (Spratt *et al*. 2021), improved soil health (Schreefel *et al*. 2020).

### Qualitative analysis

We expected that the papers could provide terms for more than one category when included in the qualitative analysis: (i) animal welfare; (ii) human welfare; and (iii) environment conservation.

**Figure 1.**
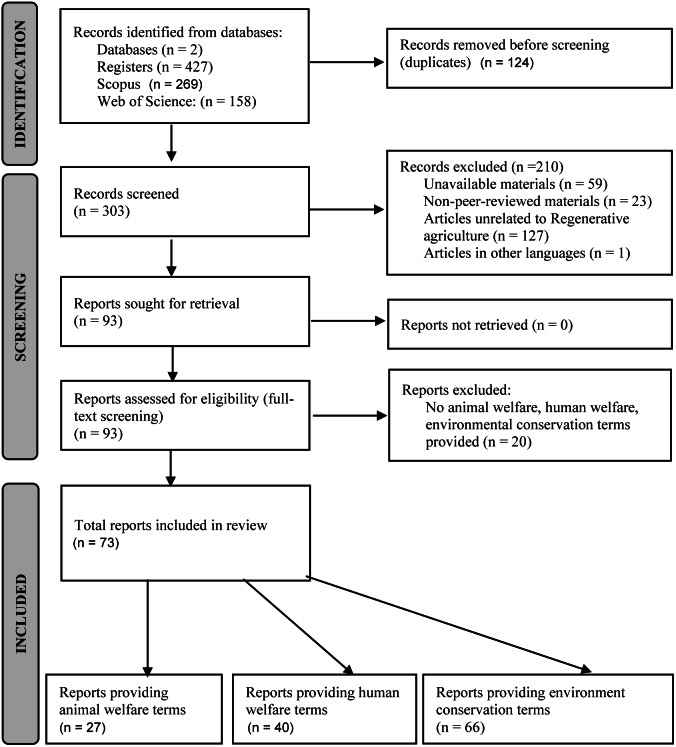
Flow chart for the identification of studies via databases and records. Adapted from PRISMA (Preferred Reporting Items for Systematic Reviews and Meta-analysis) (Pahlevan Sharif *et al* 2019, Page *et al* 2021). The discrepancy between the number of materials included by One Welfare categories (animal welfare, human welfare, and environment conservation) and the total number of materials included for qualitative analysis is explained by some materials providing terms for more than one One Welfare category.

The database search gave 427 results to our search terms combined. We screened these papers’ abstracts, titles, and keywords (no automation tools) and kept 93. The decision was made based on eligibility criteria, excluding duplicates, unavailable materials, non-peer-reviewed materials, articles in languages other than English, and papers unrelated to regenerative agriculture (Figure 1). We screened the full text of the 93 papers and excluded 20 that did not provide any animal welfare, human welfare or environmental conservation terms. We included 73 papers in the review for further qualitative analysis. The final 73 were classified into papers that provided terms for animal welfare, human welfare, and environmental conservation. Some papers provided terms for more than one category. We found animal welfare terms in 27 papers, human welfare terms in 40 papers, and environmental conservation terms in 66 papers. The 73 selected papers covered a range of species (cattle and dairy cows, sheep, and goats), mostly using the word livestock, without specifications. The 73 papers aimed to investigate the various impacts of regenerative agriculture. The majority of the papers were based in the USA (46.2% of the total materials), followed by Australia (12.9%), Canada (9.7%), and the UK (8.6%).

We clustered the terms, created themes according to their main topic, and conducted a qualitative analysis. We used the statistical tool Atlas.ti 8 to categorise and compare terms from the qualitative analysis.

### Interconnections between One Welfare categories to depict a potential benefit for animal welfare

We conducted a comparative analysis of the primary themes about animal welfare, human welfare and environment conservation, using a systems-thinking approach and the Causal Loop Diagrams tool, following the guidelines proposed by Haraldsson (2004). Causal Loop Diagrams are visual representations of critical variables, which help explore complex scenarios by uncovering variables that previous analyses might not have considered (Schlindwein & Ison 2020). We used Causal Loop Diagrams to expose interconnections and potential cause-effect relations between the three One Welfare categories within a regenerative agriculture system. These interconnections can depict underlying entry points that could ultimately benefit animal welfare. We gathered the most relevant results from each One Welfare category’s analysis and built a Causal Loop Diagram (Haraldsson 2004). We connected potential cause-effect relations of implementing a regenerative agriculture system, combining the relevant results per each One Welfare category and appropriate scientific literature to support the connections.

## Results

### Inclusion of the One Welfare categories in peer-reviewed papers about regenerative agriculture

The results of the research and the selection process, as well as the explanation for all excluded materials are presented in Figure 1.

### Animal welfare terms in regenerative agriculture

**Figure 2.**
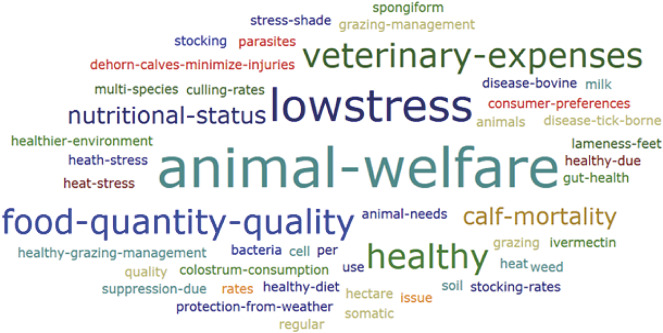
Word cloud (ATLAS.ti 8) for animal welfare terms selected from regenerative agriculture peer-reviewed papers.

We found animal welfare terms in 27 papers, with a predominance of terms such as animal welfare, low stress, veterinary expenses, food quantity and quality, healthy, nutritional status, and calf mortality (Figure 2). These terms represent the central animal welfare concepts addressed by the authors in the papers about regenerative agriculture.

**Figure 3.**
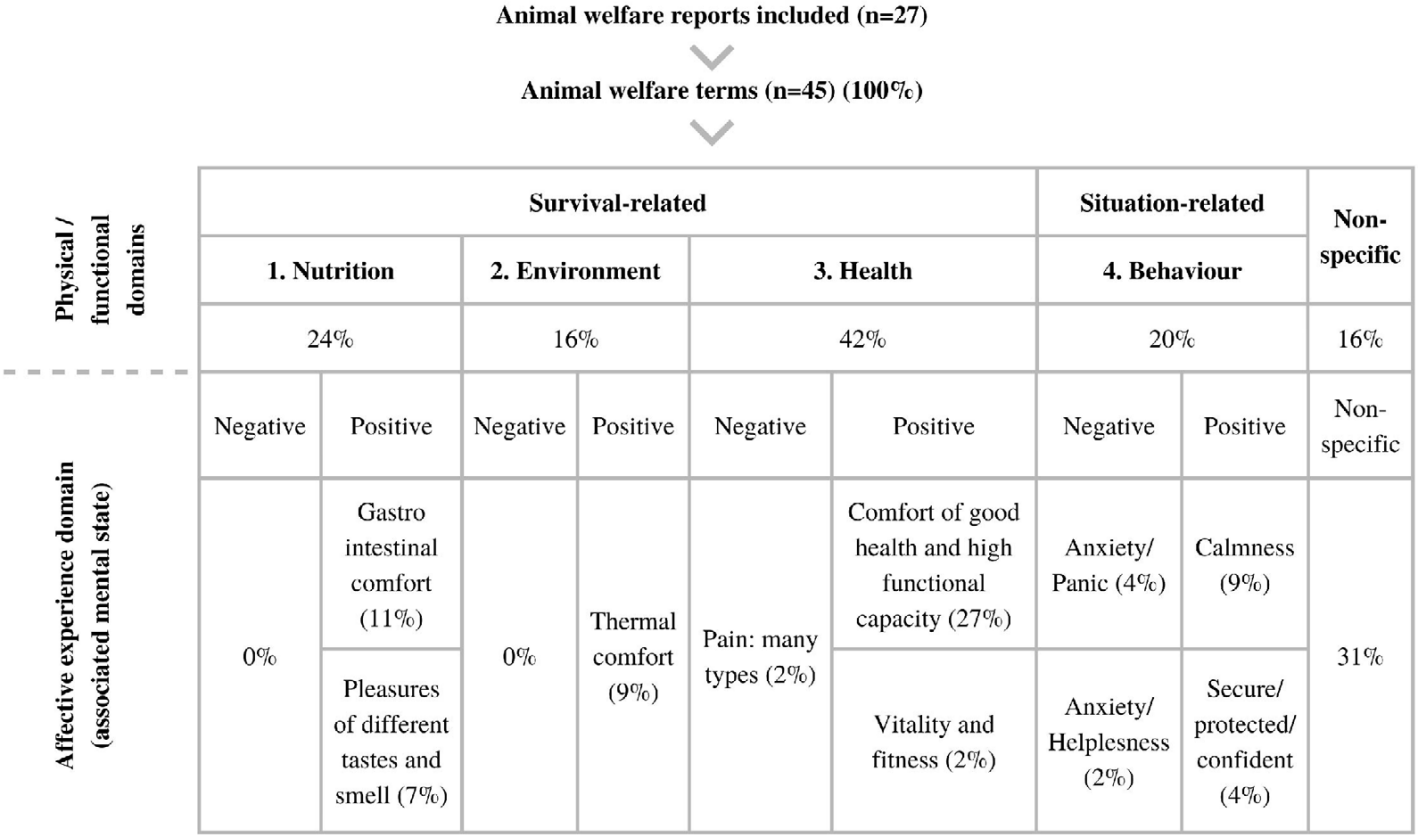
Map of the potential mental states from terms. 45 Animal welfare terms selected from 27 papers and connected to the potential mental states they could be generating. We classified these 45 terms according to the Physical/Functional Domains, 1. Nutrition, 2. Environment, 3. Health, and 4. Behaviour, and connected the animal welfare terms to their potentially generated mental state, provided by Mellor *et al* (2020).

We found that all of the animal welfare terms that we selected in the papers can be related to the physical and functional domains (Mellor *et al*. 2020), mainly to the Health domain, and less terms were connected to the Nutrition, Environment, and Behaviour domains. We found no information in the papers about the affective experience domain (Mellor *et al*. 2020). Using the Five Domains model, we built a map to highlight the potential links between the selected terms (physical and functional domains) to the associated mental state (affective experience domain) (Figure 3). We found that the majority of the selected terms could be associated to positive mental states, and only a few could be associated to negative mental states. The most frequent positive mental state was comfort of good health and high functional capacity, which is associated to the Health domain, and Mellor *et al*. (2020) define as a positive mental state, as a consequence of experiencing few or complete absence of disease, injury, functional impairment, or poisoning, and the presence of good body condition and fitness level.

### Human welfare terms in regenerative agriculture

**Figure 4.**
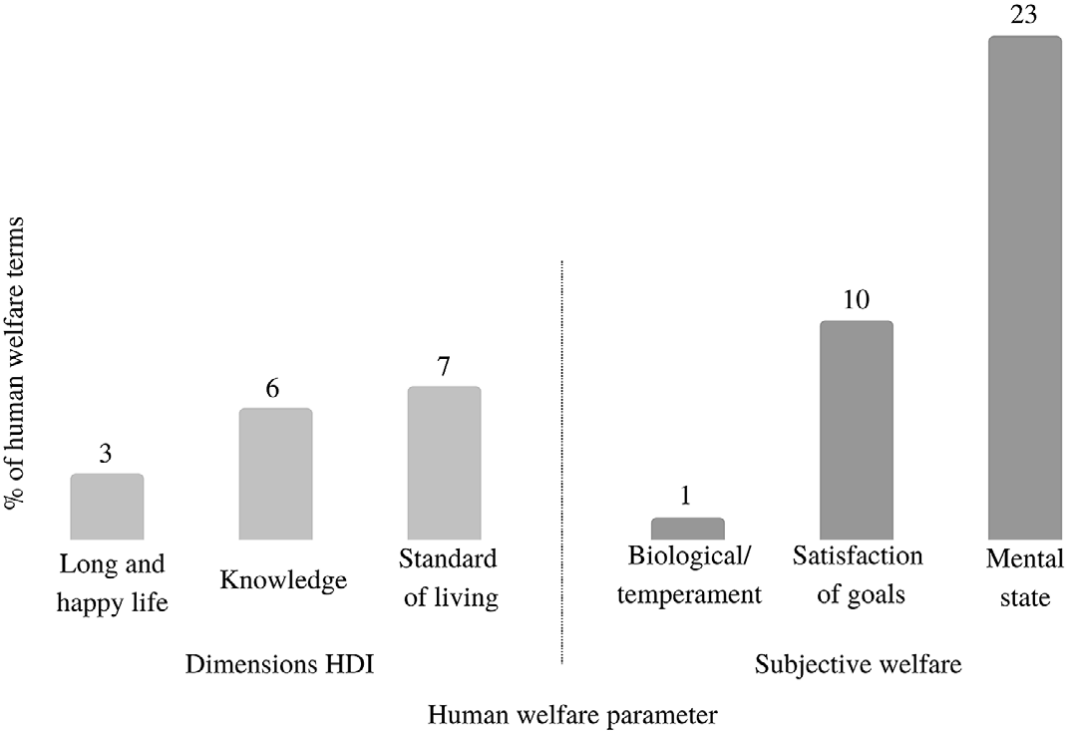
Frequency of terms that are likely to improve both hedonic and eudaimonic subjective welfare (Brown *et al* 2021). For the HDI dimensions, in the blue columns, the terms are more related to a decent standard of living. For the subjective welfare theories, in the green columns, the terms are more related to the mental-state theories (Diener *et al* 2018).

From 40 papers, we selected 116 terms relating regenerative agriculture and human welfare. After selecting the terms, we connected each term to the Human Development Index’s (HDI) dimensions from the United Nations (2020) and to a subjective welfare model (Diener *et al*. 2018). Then we connected the terms with the hedonic and eudaimonic subjective welfare (Brown *et al*. 2021), and we classified 34 terms under the label ‘likely to improve human welfare’, which means that these terms could be potentially favourable to human well-being. We looked for potential connection between these 34 terms and the hedonic and eudaimonic subjective welfare together. In Figure 4, we indicate that, regarding the Human Development Index’s (HDI) dimensions, the majority of these 34 terms were connected to a decent standard of living or financial satisfaction for the farmers engaging with regenerative agriculture. Regarding the subjective welfare model, the majority of these 34 terms were related to the mental-state theories, which describes the farmer’s perception of self-welfare when compared to a previous situation, neighbours, peers, or relatives (Diener *et al*. 2018).

### Livestock role in environment conservation

In the 66 papers for this category, we selected 202 terms that included livestock and mentioned a specific role of livestock in environmental conservation. We clustered the terms into nine themes (see Table 1).

**Table 1.** Themes developed for the environment conservation category

Theme	Explanation
1. Soil improvement	Improvements to the soil derived from an action performed by livestock in regenerative agriculture systems (e.g. trampling breaks up soil crusts)
2. Biodiversity	Biodiversity benefits as a consequence of a regenerative agriculture system (e.g. kraaling enhances palatable grass abundance)
3. Carbon sequestration	The increase of sequestered carbon as a consequence of a regenerative agriculture system (e.g. regenerative grazing increases topsoil carbon storage)
4. Environmental productive benefits	Production improvement due to an environmental enhancement in regenerative agriculture (e.g. improved forage management leads to weed suppression)
5. Land improvement	Services that well-managed livestock can deliver at a landscape scale in regenerative agriculture (e.g. ‘adaptively managing herbivores can increase the primary productivity of the landscape’)
6. Resilience	The promotion of resilient agroecosystems due to applying a regenerative agriculture system (e.g. regenerative practices can improve resilience to floods by improving the water cycle)
7. Less fertiliser and pesticides	The reduction or elimination of fertiliser and pesticides usage in regenerative agriculture systems
8. Wildlife habitat	Provision of habitat for wildlife in regenerative agriculture systems
9. Non-specific	Terms that were related to regenerative agriculture but were not specific to any theme

**Figure 5.**
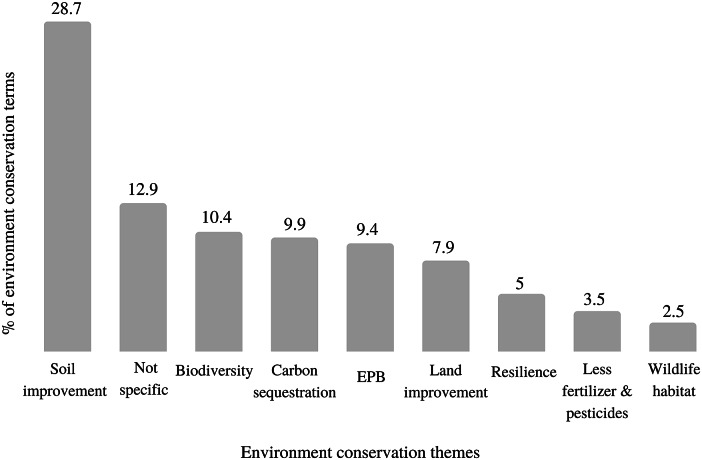
Percentage of terms related to the environment conservation themes (EPB: Environmental productive benefits).

The theme that held the majority of terms was soil improvement, implying that the primary interpretation the authors of the papers give to the role of livestock is related to improving the soil (i.e. soil health, soil fertility, and soil water holding capacity) (Figure 5). We did not find a significant number of terms related to other environmental measurements, such as water pollution/usage, land utilisation, carbon footprint, greenhouse gas emissions.

### Interconnections between One Welfare categories to depict a potential benefit for animal welfare

**Figure 6.**
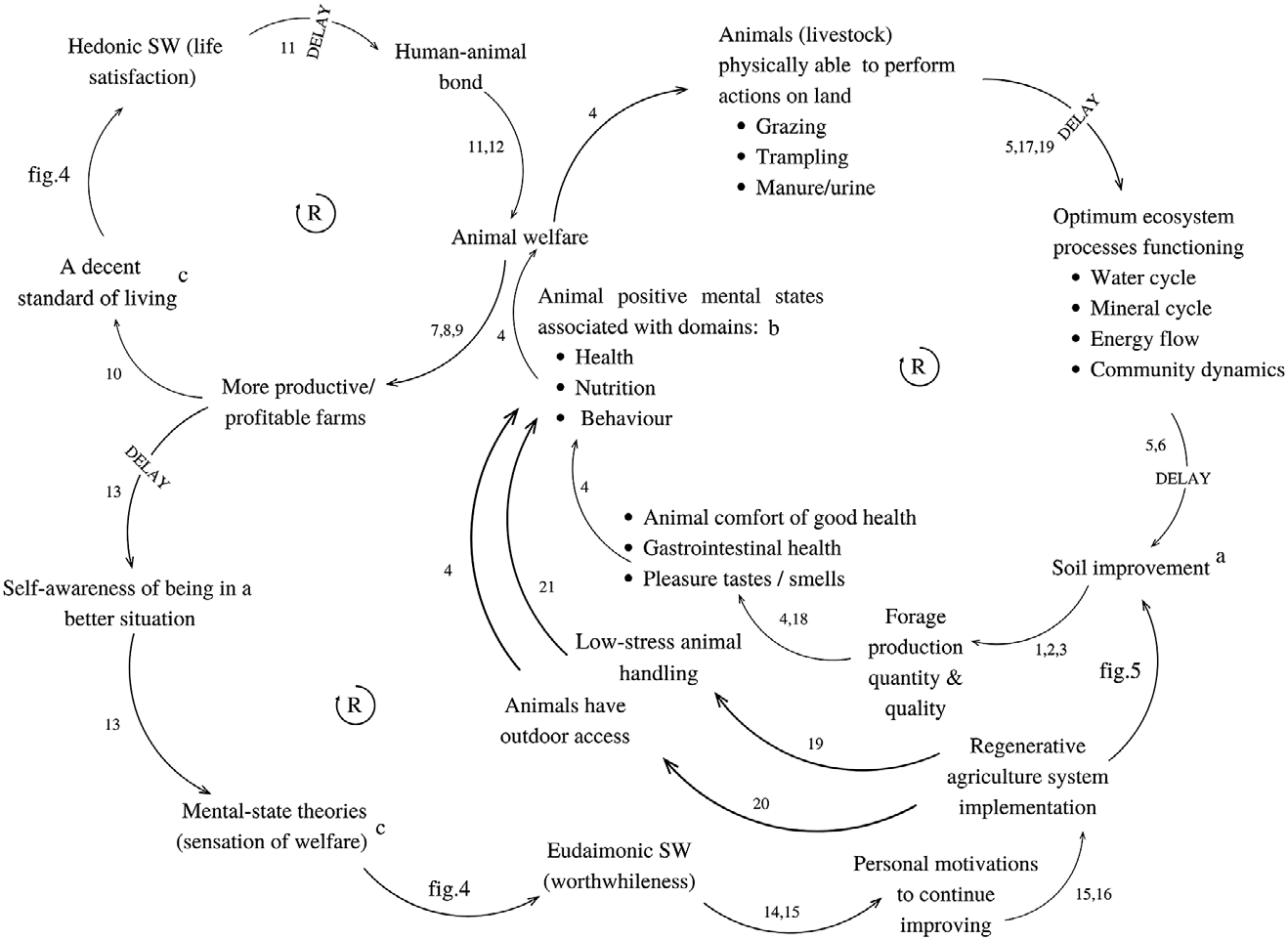
Causal Loop Diagram of the potential causes-effects of implementing a regenerative agriculture system, based on the review’s results. The word Delay represents an action from a causal relationship that takes longer than actions from other causal relationships. (1: Huruba *et al* 2018, 2: Pecenka & Lundgren 2019 3: Teague & Barnes 2017, 4: Mellor *et al* 2020, 5: Savory & Butterfield 2017, 6: Lal 2020, 7: Tarazona *et al* 2018, 8: Broom *et al* 2013, 9: Villettaz Robichaud *et al* 2019, 10: United Nations 2020, 11: Burton *et al* 2012, 12: Rault *et al* 2020, 13: Diener *et al* 2018, 14: Brown *et al* 2021, 15: Gosnell 2021, 16: Gosnell *et al* 2019, 17: Kleppel 2020, 18: Provenza *et al* 2019, 19: de Haas *et al* 2019, 20: Pinheiro Machado Filho *et al* 2021, 21: Grandin 1999) (a: resulting from the environmental conservation category’s qualitative analysis; b: resulting from the animal welfare category’s qualitative analysis; c: resulting from the human welfare category’s qualitative analysis).

With the Causal Loop Diagram (Figure 6), built from the analysis’ results for the three One Welfare categories, we present a complex scenario where we identified three reinforcement loops that emphasise potential benefits to animal welfare: (i) soil improvement provides better quality and quantity of forage (Teague & Barnes 2017; Huruba *et al*. 2018; Pecenka & Lundgren 2019), leading to animal comfort of good health and high functional capacity, a positive mental state from the Health domain; to gastrointestinal health and to pleasures of different tastes and smell (positive mental states from the Nutrition domain) (Provenza *et al*. 2019; Mellor *et al*. 2020). Healthier and well-nourished animals perform better to enhance ecosystem functioning (Savory & Butterfield 2017; de Haas *et al*. 2019; Kleppel 2020; Mellor *et al*. 2020), thus reinforcing the improvement of the soil (Savory & Butterfield 2017; Lal 2020b); (ii) when welfare improvements are delivered alongside health and productivity improvements, farms can be more profitable (Broom *et al*. 2013, Tarazona *et al*. 2020, Villettaz Robichaud *et al*. 2019), improving the financial standard of living (United Nations 2020) and, thus, farmers’ hedonic subjective welfare (Figure 4). An improvement in hedonic subjective welfare brings on better life satisfaction for farmers, which can build a positive human-animal relationship over time (Burton *et al*. 2012), thus reinforcing positive animal welfare effects due to farmers treating animals better than before; (iii) More productive and profitable farms make farmers aware that they are in a better financial situation than before, improving the eudaimonic subjective welfare related to the worthwhileness, the perception of a life worth living (Diener *et al*. 2018; Figure 4). Farmers’ awareness that their agricultural actions are worthwhile is positively related to better motivations to continue implementing regenerative agriculture, which will also reinforce the previous feedbacks (Gosnell *et al*. 2019; Brown *et al*. 2021; Gosnell 2021). Additional causal relations indicate that regenerative agriculture can contribute to the behavioural domain of animal welfare. Firstly, regenerative agriculture ensures access to outdoor pastures that meet animals’ ethological needs (Pinheiro Machado Filho *et al*. 2021). Secondly, regenerative agriculture has been associated with training practitioners in low-stress livestock handling (Gosnell 2019), which can lead to less animal stress (Grandin 1999).

## Discussion

### Animal welfare terms in regenerative agriculture

In this study, we selected information from the different authors’ interpretations of the impact of regenerative agriculture on animal welfare, human well-being, and environmental conservation; then, we interpreted and analysed this information in order to identify the potential benefits to animal welfare. Although insufficient for a comprehensive assessment of the real impact of regenerative agriculture on animal welfare, this information can illuminate the main areas that can potentially be improved by applying regenerative principles.

The selected animal welfare terms are related primarily to the Health domain and, to a lesser extent, to the Nutritional, Environmental, and Behavioural domains, indicating that the authors interpret that regenerative agriculture enhances animal health. None of the authors provided information about how they defined animal welfare or methodological details about how animal welfare was or should be assessed. Some authors concluded that regenerative practices that improve soil quality would indirectly improve the health of the animals (Sherwood & Uphoff 2000) or that animal health will improve as a consequence of mimicking the ancestral large-herds’ grazing patterns (Pecenka & Lundgren 2019). Others inferred that regenerative grazing improves animal health, thus leading to less veterinary expenses (Spratt *et al*. 2021). However, in none of these papers the promotion of other domains, rather than physical health, or positive mental states were addressed (Mellor *et al*. 2020), which would provide more information on how regenerative agriculture may improve animal welfare.

Other authors interpreted low heat stress for animals, a positive mental state associated to environment, as an outcome of regenerative agriculture (Colley *et al*. 2020; Spratt *et al*. 2021), as a consequence of providing sufficient shade to farming animals and thus protecting them against harsh weather conditions. However, providing shade is insufficient to claim that animals are in thermal comfort. Although the lack of shade could negatively influence animal welfare (Schütz *et al*. 2008), other parameters such as water availability, weather conditions, type of shade provision and structure, and social hierarchy should also be added to the reasoning (Coimbra *et al*. 2012; Deniz *et al*. 2021). Environmental assessment for better animal welfare should also consider other parameters, such as physical space, noises, odours, and light intensity, that affect a broader range of mental states (Mellor *et al*. 2020).

Some authors assume that regenerative agriculture improves animal access to sufficient, nutritious, and naturally produced food. For example, Slaughter *et al*. (2021) explain that regenerative grazing management can suppress weeds, thus improving the animal diet quality. Indeed, the suppression of weeds is an expected outcome of applying short grazing periods with high stocking rate (Savory & Butterfield 2017; Pinheiro Machado Filho *et al*. 2021). However, in the Voisin rational grazing, Pinheiro Machado Filho *et al*. (2021) explain that a balanced or better diet for animals also has to do with other factors beyond weed suppression, such as ensuring an optimal grass recovery period in the paddocks, the use of permanent multispecies swards with perennial species, the provision of fresh water, amongst others. Additionally, none of the reviewed papers addressed a connection between better food quality for animals and better food quality for humans.

The primary selected term associated with the Behaviour domain was low stress due to management. The authors did not provide in-depth details to explain exactly which type of management would lead to low stress and how. Low stress due to management related to regenerative agriculture seems to be grounded in the assumption that the system provides an improved human-animal relation, better animal handling, and lower stress as a consequence of a better environment, such as better grass, trees, and water (Gosnell 2021; Spratt *et al*. 2021). Human-animal relationships should be included in the regenerative agriculture principles, since they impact both animal welfare and human welfare (Burton *et al.* 2012; Rault *et al*. 2020). However, to conclude that regenerative management leads to a less stressful condition for animals would require a more comprehensive approach, for example, the inclusion of the principles of low-stress cattle handling (Grandin 1999), or assessments of human-animal relations, exploring the reasons that motivate the attitudes and behaviour of animal caretakers. Farmers’ welfare could be affected by life factors not related to the farm, such as family health, social support, peers’ opinion, employment conditions, the autonomy they have to express behaviours according to their attitudes (Burton *et al*. 2012; Albernaz-Gonçalves *et al*. 2021). The authors of holistic management propose, among other things, to increase the stocking rates of animals per unit of land to emulate natural large herbivore herds and thus enhance overall land performance, which could affect the relationship between caretakers and animals. In holistic management principles, it is usual to find the term ‘tool’ to refer to livestock, for example, saying that well-managed ruminants are a helpful tool to regenerate degraded land (Savory & Butterfield 2017). This utilitarian denomination may be unintended, but it might raise concerns about whether these principles explicitly consider animals as sentient beings and the need for animal welfare scientists to participate in discussions about the principles of regenerative agriculture. The regenerative agriculture principle of livestock integration should consider animals as sentient beings to minimise the risk of anthropocentric instrumentalisation of animals. Instrumentalisation is a consequence of the intensification of animal production systems, and it could risk a social devaluing of farm animal welfare (Tuyttens *et al*. 2022). However, some studies show information about regenerative farmers, primarily practicing holistic management, who declare to have improved their feelings of connection with animals, plants, and microorganisms (Gosnell *et al*. 2019, 2020). These perceptions could reflect farmers’ potential willingness or openness to explore better ways of taking care of their animals and enhance human-animal relationships.

### Human welfare terms in regenerative agriculture

The One Welfare framework indicates that the welfare of non-human animals and humans can be considered equal. The animal welfare and human welfare terms selected from the papers differ both in the authors’ interpretation and in the measurements or methods cited. This difference reflects the need for unifying the terminology across disciplines so that both animal and human welfare are treated equally in the narratives.

Most of the selected terms regarding human welfare were associated with a decent standard of living and the mental state theories. A decent standard of living, understood as an improvement in the farm’s gross income, indicates that the authors of the papers interpreted that the most relevant factor to connect human welfare and regenerative agriculture was financial. However, Sherren *et al*. (2022) indicated that farmers adopting a type of regenerative grazing can develop more than just financial welfare, but relational, physical and psychological improvements such as life satisfaction, motivation to continue with grazing, and non-traditional values.

Measuring human welfare is complex and has diverse methods and theories. The Human Development Index (as defined by the United Nations in 2020) regards human welfare as encompassing both a long and healthy life and access to knowledge. Additionally, Diener *et al*. (2018) suggest that measurements of subjectiveness are also needed to broaden the understanding of human welfare beyond physical health, knowledge, and finances. A few authors of the papers studied the relation between regenerative agriculture and subjective welfare. Brown *et al*. (2021) conducted interviews with Australian regenerative farmers, concluding that subjective welfare measures are needed in studies about regenerative agriculture, and that regenerative agriculture can be associated with high values of eudaimonic subjective welfare, which is related to personal feelings of a life worth living. Gosnell *et al*. (2019) conducted interviews with Australian farmers of holistic management to assess the factors that would improve their commitment to regenerative agriculture, concluding that there are significant traction factors associated with personal experiences that would incentivise a long-term commitment with regenerative agriculture, such as new-found humility, enthusiasm, a renewed connection to nature and community. The information provided by Gosnell *et al*. (2019) and Brown *et al*. (2021) shows that regenerative agriculture has the potential to improve other areas of human welfare besides the standard of living. Since the One Welfare framework stipulates that the welfare of human and non-human animals can be considered as equal, the efforts of scientists to develop more accurate methods to assess welfare can benefit both human and non-human animals, especially in more recently studied fields, such as mental states.

### Livestock role in environment conservation

We associated most of the terms in the environment conservation category. These terms were mainly focused on soil health and improvement. We did not find a significant number of terms related to other environmental measurements, such as water pollution, carbon footprint, greenhouse gas emissions, land erosion, overgrazing, carbon sequestration, improved biodiversity. Other environmental measurements should be included in regenerative agriculture papers that address livestock production since livestock production that is called sustainable significantly differ from industrialised systems in some of these measurements (e.g. more water pollution in industrialised operations) (Broom 2019). Regenerative agriculture should document its potential differences from industrialised systems. We found less information specifically about animal welfare and human welfare in these papers. This prioritisation of environmental terms is coherent with a global climate emergency context, where livestock production systems have been blamed for having adverse environmental effects and playing a significant role in global GHG emissions (Herrero *et al*. 2011; Cheng *et al*. 2022). The lower attention to animal welfare and human welfare may mean that these are considered less urgent than the environmental goals, or that the papers’ authors interpret that regenerative agriculture principles will naturally improve animal welfare and human welfare, without needing specific enhancing actions. In fact, several authors conclude that some environmental benefits caused by the integration of livestock, such as soil health or improvement, serve as entry points to deliver other regenerative agriculture benefits, including improved rainfall infiltration in the soil (Rhodes 2017), restoration of lost habitat and re-establishment of natural vegetation (Strauch *et al*. 2009), adaptive response to diseases (McLachlan & Yestrau 2009), the breaking up of soil crusts by trampling (Huruba *et al*. 2018), community well-being (Newton *et al*. 2020), and animal welfare (Spratt *et al*. 2021). Although some of these benefits are related to animal welfare and human welfare, they are not prioritised.

Animal welfare and human welfare are vital for any sustainable initiative (Broom 2019), given that by integrating human values, a food animal production system, like regenerative agriculture, may be more justifiable for society (Von Keyserlingk & Hötzel 2015). If researchers address this integration, regenerative agriculture may achieve more public support. However, the general public have a poor understanding of the concept of animal welfare in production systems and tend to show more concern for the welfare of animals that are considered more intelligent (Cornish *et al*. 2016). The public also criticise the industrialised profit-driven animal food production systems, where animal suffering and abuse are evident (Clark *et al*. 2016; Hötzel & Vandresen 2022). A food animal production system called regenerative, with grass-fed animals living outdoors, could then be subject to less public scrutiny about the living conditions of the animals. However, regenerative agriculture scientists and practitioners should not rely on this apparent lack of public attention and comprehensively measure society’s acceptance of regenerative systems, including, for example, more Delta Life Cycle Analyses of regenerative systems (Broom 2021), like Colley *et al*. (2020) and animal welfare measurements or data integrating biological functioning, natural behaviour, and affective states. Several elements can affect these measurements in pasture-based systems (regenerative or other) and should be addressed, such as water availability and quality, provision of shade and shelter, animal handling, protection against predators, weaning, reproductive management, disease prevention and treatment, calving, social dynamics of the herd, milking management, pasture management, infrastructure characteristics, culling methods and protocols, and human-animal bonds (Mee & Boyle 2020). Additionally, given the current process of scientifically defining the concept, regenerative agriculture is often misused and prone to greenwashing, raising red flags on many topics, including animal welfare.

### Interconnections between the One Welfare categories

The Causal Loop Diagram (Figure 6) was expected to fill the gap in the scientific literature about the potential impacts of regenerative agriculture on animal welfare, by finding positive causal relations between animal welfare, human welfare, and environment conservation. Soil improvement was the main entry point leading to potential benefits to animal welfare. The connection between regenerative agriculture and soil improvement confirms other authors’ findings that regenerative agriculture is a soil-based concept (Rhodes 2017; Elevitch *et al*. 2018; LaCanne & Lundgren 2018; Newton *et al*. 2020; Schreefel *et al*. 2020; Luján Soto *et al*. 2021). Although this review was based on peer-reviewed publications, it is challenging to determine how regenerative agriculture impacts animal welfare, so the Causal Loop Diagram was proposed to depict potential positive causal relations between regenerative agriculture and animal welfare. The diagram indicated that regenerative agriculture indirectly improves the physical health, and to a lesser extent the nutrition and behaviour components of animal welfare from three paths. Firstly, by enhancing human welfare elements, especially financial farm status and farmer’s self-awareness elements from human welfare. Secondly, by improving the soil, understanding that the soil will be the base for the rest of the ecosystem. Thirdly, by improving animal handling. However, with the results for the three One Welfare categories, we did not find relevant causal relations between regenerative agriculture and the environment domain of animal welfare, since the primary connection was thermal comfort and we found that there was no sufficient evidence in the literature to conclude that regenerative agriculture has the potential to enhance thermal comfort. The missing connections between regenerative agriculture and the environment domain reflect the challenge for a more comprehensive inclusion of animal welfare in regenerative agriculture scientific narratives.

The Causal Loop Diagram results reflect two scenarios. On the one hand, the processes that improve some components of human welfare and environmental conservation could also lead to positive animal welfare outcomes. On the other hand, failing to attend to animal welfare could result in detrimental effects for the other two categories. These interconnections align with the One Welfare framework’s central claim that the welfare of non-human animals, humans, and the environment is interconnected and should be addressed systemically.

#### Animal welfare implications

This study contributes to ensuring that animal welfare elements are present in the ongoing scientific debate about regenerative agriculture definitions. Although regenerative agriculture is a soil-based concept, it is vital to address the connections between soil and animal welfare. The soil can affect some elements of animal welfare, which need to be expanded and further studied, and failing to consider animal welfare comprehensively could have, amongst other negative impacts, detrimental effects on the soil and, ultimately, on humans. Showing these interconnections can push more key actors engaged with regenerative agriculture to place equal value on the welfare of people, animals, and the environment. The One Welfare framework succeeds in showing these interconnections, but more studies are needed to give this framework more empirical background to help its operationalisation. The findings of this study also provide orientations for animal welfare and regenerative agriculture researchers on pursuing common goals and work for better animal welfare in regenerative agriculture systems.

## Conclusion

Our main conclusions are that peer-reviewed publications exclude fundamental elements for a comprehensive understanding of animal and human welfare. While the terms for animal welfare are focused on physical health and, to some extent, nutrition and behaviour, there is a lack of terms related to needs, stress, suffering, and pleasure that could help uncover the extent of animal welfare representation in regenerative agriculture systems. Therefore, the findings provide insufficient information to determine how regenerative agriculture impacts animal welfare. We found that the selected animal welfare terms were only possible to be associated to the physical functional domains, specially to the health domain, which exposes the need to expand the study of animal welfare beyond a main focus on animal health and to include animal welfare as an integral part of regenerative agriculture. The selected human welfare terms were associated primarily to financial welfare, reflecting that the papers do not consider the welfare of non-human animals and humans as equal. A more comprehensive assessment of human welfare could benefit non-human animals and humans. In the Causal Loop Diagram, we depicted enough interconnections between the One Welfare categories to give light to further regenerative agriculture research. The latter should focus on elucidating the set of regenerative principles that could lead to better animal welfare, actively or passively, through improving human welfare and environment conservation.
